# Arsenicum-album

**Published:** 1895-08

**Authors:** 


### HOMŒOPATHIC PHYSICIAN,

A MONTHLY JOURNAL OF

HOMŒOPATHIC MATERIA MEDICA AND CLINICAL MEDICINE.

"If our school ever gives up the strict inductive method of Hahnemann, we are lost, and deserve only to be mentioned as a caricature in the history of medicine."—CONSTANTINE HERING.

Vol. XV. AUGUST, 1895. No. 8.

### EDITORIAL.

ARSENICUM-ALBUM is one of the most remarkable remedies in the homœopathic materia medica. It is one that, like Aconite, has been much abused, because given recklessly in every case of diarrhœa that occurs, just as Aconite has been given in every case of fever. Yet as was said in these pages of Aconite in fever, Arsenicum "is not the most frequently indicated remedy" in diarrhœa. No derangement of the human system rewards the careful practitioner who minutely and accurately differentiates the symptomatic indications, and prescribes thus the closest simillimum, like diarrhœa. No derangement like diarrhœa so quickly, surely, and completely responds to the simillimum, nor so certainly and vividly brings out the contrast in the condition of the patient "before and after taking" the remedy. But the gratifying result here indicated is not experienced by those prescribers who persistently give Arsenic for every case of diarrhœa that is brought to their attention, simply because it is diarrhœa and because they wish to save time and labor in studying. Perhaps the best book on the treatment of diarrhœa ever given to the profession is the remarkable monograph of Dr. James B. Bell, of Boston. This well-known volume was the means of opening the eyes of many practitioners to the necessity of careful discrimination, and by the vivid portrayal of these differences, enabled so many perplexed doctors to perform brilliant cures.

Another authority who has helped the cause along the same lines as Dr. Bell was the late Dr. P. P. Wells, of Brooklyn, who wrote a remarkable monograph upon diarrhœa, which was published as a supplement to THE HOMŒOPATHIC PHYSICIAN some years ago. Unfortunately this valuable book is now out of print. Both the works above referred to have had an immense influence in checking the tendency to routinism in the treatment of diarrhœa, and thus have given a clearer insight into the actual character of the homœopathic system.

Turning now to the notes of Dr. Lippe's lectures, we find that the first comment made by the lecturer upon Arsenic was with reference to its restlessness. The patient wishes to go from one bed to another and from one room to another. He will try every room and every bed in the house. He may walk the floor for a few minutes, just from restlessness.

Patients sometimes rise from bed during the night and walk the floor to relieve pain. This is not a clear Arsenic indication. The best remedy for this condition is Magnesia-carbonica. The Magnesia patient must get out of bed and walk the floor *to get relief from the pain.*

The Editor, by remembering this difference between Arsenic and Magnesia-carbonica, was enabled, within the last two weeks, to make a brilliant cure of intense backache located in the lumbar region and having a burning, stinging character. The sufferer who, by reason of the severity of the pain, was prevented from sleeping all night, said that she walked the floor all night. Misapprehending the meaning of the symptom and having some other symptoms to influence him, the Editor prescribed Arsenic with only partial and brief amelioration. When she endured a renewal of the attacks, it was made apparent that the walking about was in order to get relief. Magnesia-carbonica was then given with almost instant and permanent cure.

Some years after the lecture upon Arsenicum, Dr. Lippe in conversation with the Editor, referred again to this symptom of walking about, and again made clear the difference, as above stated, between Arsenicum and Magnesia-carbonica. He referred especially to a case of toothache, on the right side of the jaw, with beating or pulsating pain extending up the right side of the face into the cheek and jaw-bone, over the eye, and into the neck. The patient was compelled to walk the floor in order to relieve the pain and this suggested Magnesia-carbonica, which, being given, was followed by immediate relief.

The restlessness of Arsenicum, we will repeat, drives the patient out of bed and from one room to another. This is very different from the restlessness of Aconite, or of Apis, or of Belladonna. This restlessness was clearly defined in the editorial on Aconite in this journal last September, and in the Apis editorial in the March number.

The desire to go from one bed to another, is similar to Hyoscyamus. The characteristic restlessness of Hyoscyamus, however, is a desire to get out of bed and run away, or he asks to go home when he is already in his own house, and then tries to get out of bed. Of course this is a delirious patient. Bryonia also has this kind of restlessness, according to Bell on Diarrhœa. The Sepia patient also gets restless and *fidgety*, and wants to go from one bed to another.

Under Kali-carbonicum the patient must get up and walk about, on account of sharp stitching pains shooting from the loins to the buttocks, and occurring at three or four o'clock in the morning.

Capsicum, at night, can't get a position in which he can lie still a minute. This will remind the reader of the Eupatorium-perfoliatum patient, who can't keep still though he has a great desire to do so.

To the above the Editor adds the following indications:

Carbo-vegetabilis, restlessness and anxiety worse from four to six P. M.

Ammonium-carbonicum, restlessness of the legs. China also has restlessness of the legs; must move them and draw them up.

A number of remedies have restlessness, characterized by the patient throwing himself about the bed. Here are some of them:

Kali-jodat., restlessness; patient throws himself about the bed.

Hepar, the child throws himself about the bed unconsciously.

Ignatia, the patient throws himself about the bed wildly.

Jatropha, restlessness from pain; and the patient writhes about the bed from pain.

Calcarea-carbonica, the patient throws himself about the bed all night, with snoring, groaning.

Iodine, sits up in bed and then throws himself upon it.

Platinum, restlessness with colic; turning in every possible direction for relief.

Hydrocyanic-acid, frequently sits up in bed, gazes vacantly about him for a minute, and then throws himself forcibly down on the pillow, or flings himself upon the bed from one side to the other.

Hyoscyamus, lies on the back; sits up suddenly, and then lies down again.

Argentum-nit., must rise from bed on account of throbbing in the head.

Sepia has restlessness of legs and feet. This reminds us of Zinc.

Zinc. has restlessness of feet. "Fidgety feet." Restlessness of the legs and feet may therefore be found under four remedies, as follows: Ammonium-carb., China, Sepia, and Zinc.; but Zinc. and Sepia have specifically "fidgety feet."

This symptom of Zinc. reminds the Editor of a case of abscess of the temporal bone occurring in a child seven years old, which he had been called to treat. The abscess was in the mastoid cells. It had begun with severe pain in the left ear, and was treated by another physician, who inserted a probe in the expectation of finding a foreign body in the canal. The pain caused was so intense that the child nearly went into convulsions, and refused to again see the doctor. The writer being called to the case found an extensive abscess back of the left ear. The patient was lying on her right side, keeping very still and groaning loudly. She utterly refused to allow of any examination. She kept her feet in constant motion, shoving them past each other continuously. This suggested Zinc. and as there was great scarcity of symptoms in any event, it was decided to give Zinc. in the 200th potency. The effect was immediate and most gratifying. The pain ceased, and the restlessness of the feet disappeared also. Shortly afterward the abscess discharged into the auditory canal, and the child got well. A physician of the other school was asked what he would have done in such a case. He said the abscess suggested the possibility of dangerous inflammation of the meninges of the cerebellum; that he would have trephined the bone, and even then would have had small hopes of saving the child's life.

There is not space enough to continue comments on Arsenic in this number. But the earnest seeker after knowledge of materia medica will perceive that there lies in this article a suggestion of how he can improve his own store of information. Let him begin with the first remedy mentioned in the *Materia Medica*, and note the kind of restlessness; then proceed to the next and the next, and so on to the end. Let him write down all the symptoms of restlessness he can find, and, of course, adding the name of the remedy. He will then observe that there are several different phases of restlessness; that the remedies have much in common, and some have striking individualities. Let him group the remedies accordingly, and he will then have a magazine of information which will be of inestimable value to him in prescribing, and which he will not willingly part with if he could not duplicate it.

